# Bleeding air ambulance patients: an audit of tranexamic acid use

**DOI:** 10.1186/1757-7241-23-S2-A2

**Published:** 2015-09-11

**Authors:** Adam Chesters, Ainsley Heyworth, James Arthur

**Affiliations:** 1 Essex and Herts Air Ambulance Trust, Earls Colne, United Kingdom

## Background

Essex and Herts Air Ambulance Trust (EHAAT) teams have carried tranexamic acid (TXA) on all missions since 2012. The CRASH-2 trial demonstrated a reduction in all-cause mortality in trauma patients thought to have significant haemorrhage.

## Methods

A retrospective audit was undertaken of all EHAAT missions during 2013. All cases where the patient was classed as ‘positive’ by the Regional Major Trauma Network triage tool (MT+ve) were included. The primary outcome was the administration of the first bolus dose of 1g TXA (or paediatric appropriate dose). Compliance was defined as ‘patients thought to have significant haemorrhage received TXA’. Compliance with the local ambulance service guidelines (trauma patients with hypotension or tachycardia) was also reviewed.

## Results

84 patients were identified as MT+ve, and of these 15 received TXA. There were 8 patients documented as having significant haemorrhage. All of these patients received TXA. Blunt trauma accounted for all but 2 of the patients. The mean initial pulse rate of those patients who received TXA was 107 beats per minute (95% CI =/- 19.9) and the mean pulse rate of those patients who did not receive TXA was 87 beats per minute (95% CI +/- 6.2) (p <0.01). The mean first recorded systolic blood pressure was 101 mmHg (95% CI +/- 15.3) for those patients receiving TXA vs. 128 mmHg (95% CI +/- 6.6) for those who did not (p <0.01).

## Conclusions

We have demonstrated that 100% of patients received TXA if attended by our service and thought to be significantly bleeding following trauma. We have also demonstrated alignment with the local ambulance service guidelines pertaining to the use of TXA. Further clarification is required around the indications for TXA in all trauma patients, and EHAAT requires a specific standard operating procedure to ensure that practice is evidence-based and effective.

